# Angiotensinogen Gene Transcription in Pulmonary Fibrosis

**DOI:** 10.1155/2012/875910

**Published:** 2012-02-20

**Authors:** Bruce D. Uhal, My-Trang T. Dang, Xiaopeng Li, Amal Abdul-Hafez

**Affiliations:** ^1^Department of Physiology, Michigan State University, 2201 Biomedical and Physical Sciences Building, East Lansing, MI 48824, USA; ^2^Department of Internal Medicine, University of Iowa, Iowa City, IA 52242, USA; ^3^College of Pharmacy, Misr International University, Cairo 11331, Egypt

## Abstract

An established body of literature supports the hypothesis that activation of a local tissue angiotensin (ANG) system in the extravascular tissue compartment of the lungs is required for lung fibrogenesis. Transcriptional activation of the angiotensinogen (AGT) gene is believed to be a critical and necessary step in this activation. This paper summarizes the data in support of this theory and discusses transcriptional regulation of AGT, with an emphasis on lung AGT synthesis as a determinant of fibrosis severity. Genetic data linking AGT polymorphisms to the severity of disease in Idiopathic Pulmonary Fibrosis are also discussed.

## 1. Introduction

Pulmonary fibrosis results from injury to the lung and an ensuing fibrotic response that leads to thickening of the alveolar walls and the obliteration of alveolar air spaces. If the etiology is unknown, the condition is designated as idiopathic pulmonary fibrosis (IPF) [1]. There are also several groups of xenobiotics or environmental toxins known to cause pulmonary fibrosis, for example, the antineoplastic agent bleomycin, the class III antiarrhythmic agent amiodarone, gamma-irradiation, silicon dust, and asbestos [2]. The main histological features of the fibrotic lung are persistent and unrepaired epithelial damage, proliferation and accumulation of fibroblasts and myofibroblasts, and increased collagen deposition [3]. This section will discuss evidence that lung-derived angiotensinogen (AGT) plays an important role in lung fibrogenesis.

## 2. Lung-Derived AGT in Lung Fibrogenesis

Numerous studies support the existence of “local” angiotensin (ANG) systems in various organs and tissues. For example, the ANG II concentrations in the interstitial compartment of heart and eye were found to be 5–100 fold higher (about 50–500 pM) than that in plasma (~5–10 pM) [4, 5]. The higher interstitial levels of ANG II compared to the circulating level could not be explained by diffusion and/or receptor-mediated uptake of circulating angiotensin II. These results thereby suggest that tissue angiotensin II is largely, if not completely, synthesized locally. Furthermore, cultured cells from various organs including heart [6], vascular endothelium [7], brain [8–10], and lung [11] were shown to express the ANG system components such as AGT, ANG II, and their corresponding converting enzymes and angiotensin receptors. In contrast to the classical endocrine “renin-angiotensin-aldosterone system” (RAAS) in which the octapeptide angiotensin II is enzymatically cleaved from AGT by the actions of renin and angiotensin-converting enzyme (ACE), local angiotensin systems are categorized as either “intrinsic” (independent of the endocrine RAAS) or “extrinsic” (relying on the endocrine RAAS for some of its components). An important distinction between the endocrine and local tissue ANG systems is the fact that many local ANG systems have been shown to be independent of renin and ACE, but rather depend on other enzymes such as cathepsin D, tonin, cathepsin G, or chymase, for the enzymatic conversion of AGT to ANGII. This concept as it applies to the lung will be discussed further below; in light of the discussion to follow, in this paper the local pulmonary system described here will be referred to as an “ANG system” rather than an “RAAS” because it is not dependent on either renin or aldosterone, at least as far as our current knowledge has elucidated. First, however, the evidence that lung-derived angiotensinogen is involved in pulmonary fibrosis will be described.

 It has been shown that two cell types in the lung produce AGT: injured alveolar epithelial cells (AECs) and myofibroblasts. Primary cultures of AECs exposed to apoptosis inducers synthesize and secrete AGT and the processed peptide ANG II in response to Fas ligand [12], TNF-alpha [13], or bleomycin [14]. Primary cultures of myofibroblasts isolated from fibrotic human lungs (IPF biopsies) also expressed AGT mRNA and expressed and secreted the AGT protein as well as the processed peptide ANGII [15], strongly suggesting that human lung myofibroblasts synthesize AGT in the intact lung. Studies of myofibroblasts isolated from the heart showed that these abnormal cells express the aspartyl protease cathepsin D [16], which, like renin, also cleaves AGT to ANGI albeit at a lower pH optimum; this topic will be discussed further below.

In addition to these cell culture studies, there is *in viv*o evidence demonstrating the existence of lung-derived AGT. Endogenous AGT mRNA within lung tissue (perfused immediately before harvesting to remove blood) was upregulated *in vivo* as early as 3 hours after bleomycin instillation into rat lungs; this was demonstrated by RTPCR, *in situ* hybridization and immunohistochemistry. In the rat model, AGT mRNA and angiotensin peptides were localized in alveolar wall cells within the alveolar corners, the location of type II pneumocytes. These markers also colocalized with alpha-smooth muscle actin (*α*-SMA), the standard marker of lung myofibroblasts [17]. Those data were the first to suggest that bleomycin activated the production of the local angiotensin systems *in vivo*. Moreover, they were consistent with the previous finding of epithelial cell death in the vicinity of alpha-SMA-positive myofibroblasts in fibrotic human lung [18]. In light of the earlier demonstration that ANGII is an inducer of apoptosis in AECs [19], this finding thus supports the theory that alveolar epithelial death adjacent to myofibroblasts is caused, at least in part, by the ANG peptides they release [15]. Furthermore, in biopsy specimens obtained from the lungs of patients with Idiopathic Pulmonary Fibrosis (IPF), AGT mRNA and protein were 21-fold and 3.6-fold more abundant, respectively, in IPF biopsies relative to biopsies of normal human lung [20]. The data from the IPF lung biopsies are consistent with data from bleomycin-induced rat lung fibrosis models [17].

## 3. AGT Transcription in the Lung as the Rate-Limiting Step of Pulmonary Angiotensin II Generation in Bleomycin-Induced Pulmonary Fibrosis

Recently our research group demonstrated that intratracheal administration of antisense oligonucleotides against AGT mRNA prevented bleomycin-induced lung cell apoptosis and lung fibrogenesis by blockade of the synthesis of lung-derived AGT [17]. Two important aspects of that study were the findings that fluorescently tagged antisense, when administered intratracheally, were taken up exclusively by the lungs, but less than 5% was taken up by liver or kidney. Consistent with that finding, the antisense oligos completely blocked the bleomycin-induced increase in lung tissue AGT, but had no effect on serum-borne AGT or circulating ANGII. Those data strongly implicated organ-specific transcription of lung-derived AGT as a critical determinant of lung fibrogenesis.

Investigations of primary cultures of AECs and lung explant cultures demonstrated some of the many factors capable of inducing AGT expression in lung tissue. AGT mRNA expression in primary AECs and the human AEC cell line A549 is upregulated by, and functional AGT mRNA is* required* for, the apoptotic response to Fas L, TNF-*α*, bleomycin, and amiodarone; this was shown by the demonstration that the apoptosis of primary AECs and A549 cells caused by these agents was blocked by the same AGT antisense oligos used in the preceding paragraph, but not by scrambled-sequence control oligonucleotides of the same base composition as the antisense [21]. In another model of cultured lung explants, in which the “endocrine” RAAS is not active, bleomycin-induced collagen accumulation *ex viv*o also required AGT gene expression; the AGT antisense oligos also blocked collagen deposition in this model [17].

Other considerations support the notion that local transcription of AGT, as well as converting enzymes synthesized locally rather than derived from the “endocrine” RAAS, comprises an “intrinsic” ANG system that is upregulated in lung injury but independent of the endocrine RAAS. In the whole animal model, circulating levels of angiotensinogen are known to be approximately equal to the Michaelis Constant (*Km*) of renin for its substrate (about 1 uM) [22]. Therefore, the rate of angiotensin II synthesis can be regulated by changes in angiotensinogen levels. Since the normal concentration of AGT is near the *Km *for its cleavage by renin [22], one might expect any change in AGT levels to be accompanied by parallel changes in the formation and actions of ANG II. Published findings support this concept; for instance, transgenic mice overexpressing the rat AGT gene are hypertensive [23] and mice underexpressing the AGT gene are hypotensive [24]. In addition, for any given level of renin activity, angiotensin II synthesis can be altered by changes in the concentration of available AGT [25]. For example, upregulation of AGT in tissue can alter tissue concentrations of angiotensin II, particularly, in tissues which are not subjected to systemic short-loop or long-loop feedback control [25]. In the bleomycin-induced pulmonary fibrosis model, AGT expression is rapidly upregulated in blood-free lung tissue. This elevation of local AGT increased the lung tissue ANG II concentration. Furthermore, intratracheal delivery of antisense oligos against AGT mRNA blocked apoptosis of alveolar epithelial cells and subsequent collagen deposition, both of which are consistent with the result that the antisense oligos decreased local ANG II generation [17].

Whether the circulating ANGII contributes to the local pulmonary ANGII that mediates lung fibrogenesis is unlikely, for several reasons. First, the concentration of ANGII in the serum is extremely low, on the order of 1–5 pM, due to the many peptidases in the serum that rapidly degrade the octapeptide [26]. Measurements of tissue ANGII by Marshall et al. [27] suggest that lung tissue ANGII concentration is much higher, especially after lung injury that activates the local lung ANG system. Moreover, uptake from the circulation can be quantified by measuring steady-state tissue and plasma levels of 125I-labelled ANG I and II during 125I-ANGI and II infusion. Results obtained from pigs [4, 28] showed that I25I-ANG II, but not 125I-ANG I, accumulated in tissues like heart, kidney, and adrenal gland. The absence of significant tissue 125 I-ANG I accumulation indicated that the presence of tissue ANG I, at least in those organs, cannot be attributed to uptake from circulation. Accumulation of 125 I-ANG II at tissue sites was largely prevented by pretreatment with an AT1 receptor antagonist [29], suggesting that uptake of ANG II is mediated via AT 1 receptor-dependent endocytosis. It is unlikely that AT2 receptors play a role in this process since AT2 receptors do not internalize ANG II [30]. Similar conclusions about that AT1 receptor mediates internalization of ANG II were drawn from ANG II infusion studies in rats [31, 32]. However, comparison of the 125 I-labelled and endogenous angiotensin levels revealed that, despite the significant uptake of 125I-ANG II in various tissues, the majority (>90%) of tissue ANG II is not derived from circulation. Instead, it is synthesized from locally generated ANG I [33]. In the lung ANGII uptake may not exist as treatment with losartan increased rather than decreased pulmonary ANG II concentration [27].

Moreover, several lines of evidence argue that enzymes other than renin and ACE are involved in local ANGII generation from lung-derived AGT, in contrast to the “endocrine RAAS” which is dependent on these two enzymes. First, the ANGII-dependent apoptosis of alveolar epithelial cells that results from exposure to bleomycin was shown to require cathepsin D, an aspartyl protease that serves the same function as renin, although at a lower (~6.0) pH optimum [34]. The induction of apoptosis both activated and upregulated cathepsin D and AGT within the target cell itself and also promoted their release into the extracellular space [ibid]. The latter may be particularly important in light of the relatively low pH within the aqueous extracellular environment immediately above the type II alveolar epithelial cell *in vivo*. This region, known as the “alveolar hypophase” or subphase, is an aqueous layer immediately above the epithelial cell but below the surfactant phospholipid monolayer. Experiments with microelectrodes have shown this microenvironment to have a pH of about 6.6–6.9, due to proton pumps and Na+/K+-ATPases on the AEC which function in unidirectional energy-dependent water movement out of the alveoli [35]. Thus, AEC-derived AGT and cathepsin D are released from AECs after lung injury into a microenvironment with a pH favorable for cathepsin D-mediated cleavage of AGT to ANG I. Indeed, even if renin were sequestered from the serum, the low pH in this microenvironment would be predicted to favor cathepsin D activity over that of renin. In addition, cathepsin D was shown to be upregulated in fibrotic human lung [36] and in rat lung epithelial cells undergoing apoptosis *in vitro* in response to advanced glycation end products [37].

Likewise, inhibitors of chymase, one of the other enzymes known to have the same ability as ACE to convert ANGI to ANGII, have been shown to inhibit paraquat-induced lung injury and fibrosis in mice [38] and bleomycin-induced lung fibrosis in hamsters [39]. Thus, the presence of renin or ACE in the local pulmonary ANG system is not necessary for conversion of AGT to ANGII. Taken together, our results and those of other groups strongly suggest that the local ANG system described here does not require enzymes from the endocrine RAAS and that expression of AGT *de novo* in the lung is the most likely rate-limiting step for angiotensin II generation within the extravascular compartment of the lung parenchyma.

## 4. Regulation of Angiotensinogen Gene Expression

### 4.1. The Angiotensinogen Gene

The human angiotensinogen gene is located on chromosome 1q42-q43 and contains 5 exons/4 introns showing organization similar to other serine protease inhibitors (serpins) superfamily to which AGT belongs. The human AGT gene encodes the human angiotensinogen molecule, an *β*_2_-globulin protein (485 amino acid), with a molecular weight of about 61 kDa. Exon 1 of the human AGT gene encodes for a short 37 bp 5′ untranslated tract, with the second exon encoding the initiation methionine, signal peptide, and the majority of the mature protein [40, 41]. AGT abundance is regulated at the transcriptional level through hormonal and cell-type-specific regulators.

### 4.2. Constitutive AGT Gene Expression

Constitutive human AGT gene transcriptional control has been studied extensively in liver [40]. The human angiotensinogen core promoter element 1 (AGCE1) site, located between the TATA box and transcription initiation site, was shown to be a critical regulator of AGT transcription by binding to human angiotensinogen core promoter binding factor 1(AGCF1) in the human hepatoma cell line HepG2 cells [42]. USF1 has been identified as a component of AGCF1 [43]. AGCE1 was also shown to be required for the activity of an upstream AGT enhancer, ATF-like element (ALE) [44]. In addition to AGCE1, Yanai et al. also describes two AGCE2 sites, located upstream and downstream of the transcription initiation site that show a possible cell-type-dependent function [45]. The hepatocyte nuclear factor 4 (HNF4) was also shown to potentiate human AGT (hAGT) promoter activity in HepG2 cells [46].

Other cell type-dependent elements controlling AGT transcription are direct repeat sequences (DR) in AGT promoter that contribute 50 or >95% to AGT transcription in liver or kidneys, respectively, whereas the same sequences are not required in the heart and brain [47], and a 3′-downstream enhancer that binds an AP-3-related factor and human angiotensinogen enhancer factor-1 (hAEF-1) [48]. In kidney cells, intrarenal AGT expressed in renal proximal tubular cells was shown to be regulated by nuclear factor-*κ*B (NF-*κ*B) binding to an NF-*κ*B-binding site in the hAGT promoter region [49].

### 4.3. Regulated AGT Gene Expression in Different Tissues

Inducible human AGT transcription has been studied in response to several factors. In HepG2 cells, interleukin- (IL-) 6 induces AGT transcription via STAT3 binding to one of three acute-phase response elements (APREs) present between −350 and −122 upstream of transcription start site [50]. Recently, Jain et al. suggested that three transcription factors, glucocorticoid receptor (GR), STAT-3, and HNF-1*α*, bind to the APREs region of the hAGT gene promoter and are responsible for IL-6-induced promoter activity of this gene in liver cells [51]. Upregulation of human AGT gene transcription in Hep3B hepatocytes by interferon-gamma was shown to involve STAT1-binding motif in the AGT promoter between −271 and −279 in a mechanism separate from IL-6 upregulation of AGT by STAT3 [52].

Other inducers of AGT transcription include estrogen, through an estrogen responsive element near TATA box of the promoter [53]. In a study suggesting relation between adipose AGT secretion in obesity and elevated blood pressure, AGT gene expression and secretion in adipose tissue were found to be stimulated by cyclic AMP via increased DNA cyclic AMP-responsive element- (CRE-) binding activity [54].

 Repression of AGT gene transcription was described by Date et al. [55], where Finb, a multiple zinc finger protein, was reported to repress transcription of the AGT gene via binding of two elements in the 5′ flanking region of the human AGT promoter [56]. Expression of AGT gene was also shown to be repressed in HepG2 cells in response to bile acids via the small heterodimer partner (SHP) acting on the binding site of hepatocyte nuclear factor-4 (HNF-4) on the AGT gene promoter [56].

### 4.4. Regulated AGT Gene Expression in Pulmonary Fibrosis

As angiotensinogen is the precursor protein for the ANGII peptide, the regulation of AGT gene expression in the lung under fibrotic conditions has been studied by our research group in response to profibrotic and proapoptotic inducers. In cultured pulmonary epithelial cells, the antiarrhythmic drug amiodarone, which is fibrogenic for the lungs at therapeutic doses, induced AGT transcription through the AP-1 site present between the TATA box and transcription start site [57]. Transforming growth factor-beta1 (TGF-*β*1), the prototype mediator of extracellular matrix deposition in the lungs and other tissues [1], was found to upregulate AGT transcription in human lung fibroblasts [57]. Mechanisms for this regulation required binding of both the AP-1 family transcription factor JunD and hypoxia inducible factor-1 alpha (HIF-1*α*) to the AGT core promoter [58]; the significance of these factors, especially HIF-1*α*, is discussed further in the following paragraphs.

### 4.5. Hypoxia, Angiotensin, and Pulmonary Fibrosis

It is notable that hypoxia inducible factors have been previously suggested to be involved in the pathogenesis of lung fibrosis. In alveolar epithelial cells, hypoxia was found to induce apoptosis of primary cultures of alveolar epithelial type II cells in a manner comparable to the effects of ANGII on these cells [59]. HIF-1*α* overexpression was detected in the so-called “hyperplastic epithelium” of fibrotic lungs [60], which is found in the same regions immediately adjacent to the myofibroblast foci in which the apoptotic epithelia described above were found [18]. Moreover, the activation of HIF pathways results in epithelial-mesenchymal transition and fibrosis [61, 62]. On the other hand, in lung fibroblasts the overexpression of von Hippel-Lindau protein (pVHL) that targets HIF for degradation failed to decrease HIF signaling [63], and the TGF-*β*1 that mediates transition of fibroblasts to myofibroblasts increases the nuclear abundance of HIF-1*α* protein [58]. In light of our demonstration that the transcription of AGT in response to TGF-*β*1 is mediated by the hypoxia-signaling transcription factor HIF-1*α*, these findings suggest a molecular mechanism linking hypoxia-signaling and fibrogenic stimuli in the lungs by modulation of the levels of the ANGII peptide precursor AGT [58, 59].

## 5. SNPs in AGT Important in Organ Fibrosis

As discussed above, the severity of fibrotic diseases in the heart and kidney is influenced by ANGII. In the heart, ANGII plays a role in the hypertrophy of cardiomyocytes, fibroblast hyperplasia, and interstitial cardiac fibrosis [64]. Similar effects are also seen in the liver. The progression of congenital hepatic fibrosis is mediated through the increase of angiotensin-converting enzyme (ACE), ANGII, and TGF-*β*1 [65]. This was also reflected in the disease progression of liver injury following chronic hepatitis C infection, in which liver fibrosis severity was highly associated with genotypes leading to higher expression of TGF-*β*1 and AGT [66]. As discussed above, local expression of AGT is a requirement for the experimental induction of lung fibrogenesis [1, 16]; additionally, ANGII is a mediator of the fibroproliferative response in acute lung injury [27]

Activated epithelial cells and myofibroblasts are sources of ANGII. The profibrotic effect of ANGII is mediated through the stimulation of TGF-*β*1 and the induction of epithelial mesenchymal transition (EMT) resulting in the differentiation of epithelial cells, in addition to normal fibroblasts, into myofibroblasts. Excess deposition of collagen by myofibroblasts leads to the final result of fibrosis. Recent work showed that AGT mRNA and protein are constitutively expressed in human lung myofibroblasts in response to “autocrine loops” of TGF-*β*1 and AGT expression which drive and expand each other unless interrupted [1]. Most important for the following discussion is that the upregulation of AGT by TGF-*β*1 is mediated through the AGT core promoter, which contains three single nucleotide polymorphisms (SNPs), A-20C, C-18T, and G-6A [43, 58, 67].

In fibrosis of organs other than the lungs, interindividual variability in the progression or severity of fibrotic disease is correlated with genetic variants in AGT at those three genetic loci. In a Spanish IPF cohort, it was demonstrated that the AA genotype of G-6A SNP was significantly associated with disease progression as measured by changes in the alveolar-arterial oxygen gradient over time [68]. This same genotype was also linked to an increase in hepatic fibrosis in people with chronic hepatitis C infections and in advanced liver fibrosis in the severely obese [66, 69]. In the heart, G-6A and A-20C SNPs are associated with an increase in mean carotid intimal-medial thickening (IMT) in females [70]. In a similar manner, both of these SNPs displayed a significant relationship in liver cirrhosis in patients with chronic hepatitis B [70]. The G-6A SNP is found in partial linkage disequilibrium with A-20C and C-18T. As a consequence, a higher frequency of G-6A is found with C alleles at the −20 and −18 positions. Due to the scarcity of the C-18T genotype in the human population, G-6A is more frequently seen with A-20C.

The progression of fibrosis is exacerbated when variants in AGT are inherited in conjunction with variants in other genes. For example, the inheritance of variants in TGF-*β*1 and AGT together is associated with increased staging of hepatic fibrosis [66]. Individually, these variants are correlated with higher stages of fibrosis; however, the inheritance of both the arginine/arginine genotype in codon 25 of TGF-*β*1 and the AA genotype in −6 of AGT, together, led to more progressive fibrosis than either variant alone [ibid]. This effect was also associated with advanced hepatic fibrosis in obese patients with nonalcoholic fatty liver disease [69]. The interaction of A-6C of AGT with the I allele of ACE leads to an increase in the mean IMT in the population as a whole [70].

Based on these studies, it is hypothesized by our research group that the progression and/or severity of collagen deposition in IPF may be predicted by a risk haplotype in the AGT gene. The theorized risk haplotype that will be found to predict IPF severity is CCA (−20, −18, and −6, resp.). In addition, it is predicted that inheritance of this variant with the arginine/arginine genotype in codon 25 of TGF-*β*1 and/or the D allele of ACE, which also has been associated with more severe lung fibrosis [71], will further intensify the progression or severity of IPF. Investigations of these hypotheses are currently underway.

## 6. Clinical Significance

Given the widespread use and relative safety of inhibitors of the ANG system in cardiovascular diseases, the potential of these inhibitors in the therapeutic management of patients with lung fibrosis is of high interest. Initial attempts to find therapeutic benefit of ACE inhibitors through retrospective analyses of clinical data from individuals receiving ACE inhibitors for other comorbid conditions did not find therapeutic improvement of lung fibrosis [72, 73]. However, these studies were not designed as prospective tests of lung fibrosis alone nor were the subjects chosen randomly. Moreover, the above discussion of ACE-independent pathways of ANGII formation in local tissue ANG systems argues against the prospect of therapeutic benefit through inhibition of ACE. Indeed, a recent study of the counter regulatory ACE-2/angiotensin 1-7/mas axis [74] has shown that ACE inhibitors might, at least theoretically, prove to be counterproductive through their potential reduction of the inhibitory peptide angiotensin 1–7. This heptapeptide, which is the product of ANGII degradation by ACE-2, inhibits the actions of ANGII and is normally present in much higher levels than the profibrotic ANGII [ibid]. On the other hand, significant published data support the potential efficacy of AT1 receptor antagonists in animal models (see previous discussion), and several clinical trials of AT1 receptor blockers in IPF are currently underway [75]. An alternate therapeutic strategy might be the reduction of lung-specific AGT transcription through antisense oligonucleotides delivered specifically to the lungs [17].

## 7. Summary and Conclusions

Activation of the local ANG system in lung tissue is now known to be a required event in the pathogenesis of experimental lung fibrosis. Data from lung biopsies obtained from patients with Idiopathic Pulmonary Fibrosis (IPF) are consistent with the hypothesis that a local lung ANG system also is activated in human lung fibrosis. Transcription of the angiotensinogen (AGT) gene is the rate-limiting step in this activation, and antisense oligonucleotides against AGT mRNA prevent both lung epithelial cell apoptosis and the accumulation of lung collagens. Single nucleotide polymorphisms (SNPs) in the AGT gene, some of which are known to be significantly associated with essential hypertension and higher rates of AGT gene transcription, have been shown to associate with more rapid progression of IPF. It is hoped that the further analysis of additional SNPs in the AGT gene will reveal an “IPF risk haplotype” in AGT that might hold significant predictive value in the treatment of IPF and possibly other lung diseases that are influenced by lung AGT expression.
